# Complex Intramolecular Mechanics of G-actin — An Elastic Network Study

**DOI:** 10.1371/journal.pone.0045859

**Published:** 2012-10-15

**Authors:** Markus Düttmann, Markus Mittnenzweig, Yuichi Togashi, Toshio Yanagida, Alexander S. Mikhailov

**Affiliations:** 1 Department of Physical Chemistry, Fritz Haber Institute of the Max Planck Society, Berlin, Germany; 2 Department of Mathematics and Computer Science, Free University Berlin, Berlin, Germany; 3 Graduate School of System Informatics, Kobe University, Kobe, Hyogo, Japan; 4 Quantitative Biology Center (QBiC), RIKEN, Suita, Osaka, Japan; 5 Graduate School of Frontier Biosciences, Osaka University, Suita, Osaka, Japan; Koç University, Turkey

## Abstract

Systematic numerical investigations of conformational motions in single actin molecules were performed by employing a simple elastic-network (EN) model of this protein. Similar to previous investigations for myosin, we found that G-actin essentially behaves as a strain sensor, responding by well-defined domain motions to mechanical perturbations. Several sensitive residues within the nucleotide-binding pocket (NBP) could be identified, such that the perturbation of any of them can induce characteristic flattening of actin molecules and closing of the cleft between their two mobile domains. Extending the EN model by introduction of a set of breakable links which become effective only when two domains approach one another, it was observed that G-actin can possess a metastable state corresponding to a closed conformation and that a transition to this state can be induced by appropriate perturbations in the NBP region. The ligands were roughly modeled as a single particle (ADP) or a dimer (ATP), which were placed inside the NBP and connected by elastic links to the neighbors. Our approximate analysis suggests that, when ATP is present, it stabilizes the closed conformation of actin. This may play an important role in the explanation why, in the presence of ATP, the polymerization process is highly accelerated.

## Introduction

Actin is one of the most abundant proteins of the living cell. It builds part of the cytoskeleton and is involved in cell motility, transport inside the cell and muscle contraction [1]. A property of this protein, essential for its biological functions, is that it can polymerize forming long helical filaments. Actin is also an enzyme catalyzing hydrolysis of adenosine triphosphate (ATP) into its products adenosine diphosphate (ADP) and inorganic phosphate (P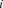). It is well known that, in the presence of ATP, the polymerization process is greatly accelerated [2], [3]. Because of its importance, extensive experimental investigations of actin have been undertaken and a large amount of data is available. By using X-ray crystallography, equilibrium structures of actin monomers (G-actin) in complexes with ADP [4] and ATP together with other proteins [5], [6] have been determined. Employing cryo-electron microscopy, the equilibrium structure of filaments (F-actin) could be identified with high resolution [7]–[11]. Fluorescence resonance energy transfer (FRET) investigations of filaments have been performed, revealing the dynamics with transitions between distinct conformational states [12]. Nonetheless, many functional aspects of actin are not yet fully understood. Particularly, this refers to the role of ATP and the effects of nucleotide binding and hydrolysis reaction. Theoretical studies of actin models should contribute to the clarification of such aspects.

In enzymes, binding of ligands and catalytic conversion reactions are often accompanied by conformational changes, so that pronounced mechanochemical motions take place inside these proteins (see, e.g., [13]–[15]). Moreover, catalytic reactions and binding or release of ligands may also be affected by the application of external strains. This has been recently directly demonstrated for myosin, which, being a molecular motor, is also an enzyme catalyzing ATP hydrolysis. Depending on the direction of the applied force, coupling of myosin to actin filaments [16] and its ADP affinity [17] could be controlled. Thus, myosin could be seen as a single-molecule mechanical sensor, with chemical events being sensitively modulated by the strains which were externally applied.

The strain-sensor behavior of myosin in response to the application of external forces to its tail was confirmed in recent theoretical investigations [18]. It was furthermore found that the protein responds by definite conformational changes to the forces applied to individual residues in the nucleotide-binding pocket (NBP) and such characteristic responses are strongly sensitive to the choice of the residues to which the perturbations are applied. Thus, the strain-sensor behavior can also underlie intrinsic mechanochemical motions in myosin, i.e. its responses to binding and detachment of ligands. In the present work, a similar investigation of mechanical properties and intrinsic responses is undertaken for G-actin.

A detailed theoretical description of mechanochemical conformational motions in proteins can be provided by all-atom molecular dynamics (MD) simulations. The difficulty encountered is that such motions are typically on the scale of milliseconds or longer, whereas, in full MD simulations, only the dynamics on much shorter time scales can be resolved. To overcome this limitation, various acceleration methods have been proposed (see, e.g., [19]–[22]) and specialized hardware for efficient MD simulations is being developed [23]–[25]. By using accelerated MD simulation methods, conformational dynamics of actin monomers has been investigated and responses due to interaction with ATP and other ligands have been analyzed. Nucleotide-dependent folding of the flexible DNase-I binding (DB) loop could be observed [26], [27]. It was demonstrated that the nucleotides induce significant local deformations in the region of the ATP binding pocket which can become partially closed [27].

An alternative to all-atom models of proteins is provided by coarse-grained dynamical descriptions. Various such descriptions have been proposed (see a review in Ref. [28]) and the G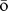-like models [29]–[32] deserve to be particularly mentioned. In the last years, elastic-network (EN) models of proteins became increasingly popular [33]–[41]. In this approach, amino acids are replaced by identical point particles and the interactions between residues are approximated by introducing elastic links between such particles, so that an elastic network is obtained. The links have different natural lengths, but, as often assumed, the same stiffness. The architecture of the elastic network, i.e. the pattern of connections between its nodes, is constructed on the basis of experimentally known equilibrium conformation of a protein. Several variants of the EN approach exist and the method known as the anisotropic-network model [42], [43] is used in our investigations.

Despite their high degree of simplification, EN models have turned out to be remarkably effective. Investigations revealed that such models can predict ligand-induced conformational changes and describe thermal fluctuations (B-factors) in many proteins [39]–[47]. While much attention has been paid to the normal-mode analysis corresponding to linearized EN models, full nonlinear equations of relaxational EN dynamics were also explored [18], [48]–[53]. It was shown that EN models can be extended to include the possibility of partial unfolding and refolding of proteins [36], [37], [54], [55]. Effects of hydrodynamic interactions with the solvent can be incorporated into the EN models [56], [57] and thermal fluctuations can also be considered in the framework of this approach. Entire operation cycles of the molecular motor hepatitis C virus (HCV) helicase [52] and of the enzyme adenylate kinase [56] could be effectively reproduced within the EN simulations.

There is one property of elastic-network models which makes them particularly appealing. Since all residues are pictured as identical point particles, irrespective of their actual chemical structure, and because interactions between the particles do not depend on the chemical nature of the corresponding residues, EN models turn out to be stripped of almost all chemical details. In this rough approximation, a protein is viewed as a mechanical object, i.e. as a complex elastic network. Therefore, by studying such models, one can investigate purely mechanical aspects of conformational protein dynamics, distinct from the chemical aspects. By using the EN approach, we have previously analyzed large-scale conformational responses of myosin-V to the application of forces to individual residues in its various functional regions, including the NBP [18]. Thus, the strain-sensor behavior in this motor protein could be demonstrated and the results of the single-molecule experiments [16], [17] could be explained. In our EN study of HCV helicase, interactions with the double-strand DNA have been taken into account and the ratchet inchworm mechanism of translocation and DNA unwinding could be directly verified [52].

Previously, conformational dynamics of G-actin was investigated through the normal-mode analysis using an all-atom description for the protein and its ligands [58]. Two slow modes, corresponding to characteristic propeller and scissor motions of the principal domains, were found. However, such normal-mode analysis is strictly applicable only for small-magnitude conformational motions, so that the linearization of dynamical equations in terms of atomic displacements is still justified. In contrast, large-magnitude mechanical motions in G-actin are considered in this study. Our analysis goes beyond the normal-mode description and is based on full nonlinear equations of the EN approach. Moreover, emergent (and breakable) elastic links are introduced into the model, so that, in addition to the equilibrium state of the network, its metastable stationary states appear. Note that a nonlinear double-well EN model has previously been employed to consider the coil-to-helix transition of the DB loop in G-actin [59].

Our aim was to systematically probe mechanical responses of the actin macromolecule and to understand internal organization of its dynamics. As we have found, two mobile actin domains are able to perform well-defined large-scale motions. They can come so close to each other that additional interactions between the residues from different domains develop; thus the actin can get locked in a metastable closed state. Furthermore, our detailed study of mechanical sensitivity of the molecule to application of various perturbations in the nucleotide-binding region has revealed that characteristic global domain motions can also be easily induced by application of only local perturbations to some selected residues in the NBP. Binding of nucleotides and the hydrolysis reaction lead to local mechanical perturbations in the NBP and, in this way, can induce characteristic large-magnitude motions of mobile domains. In the coarse-grained EN models, chemical aspects of ATP binding cannot be adequately resolved. In the future, more elaborate investigations combining the EN approach with local MD simulations may be needed to systematically study such processes. In the present study, such effects were approximately considered by placing a fictitious ligand dimer into the actual nucleotide binding pocket and by introducing elastic links between the ligand and the nearest residues. We have found that binding of a model ligand, imitating the ATP, can stabilize the closed state of actin and induce a transition to this state from the equilibrium open state of the molecule. This observation can be important for understanding the role of ATP in the polymerization process.

## Results

**Figure 1:**
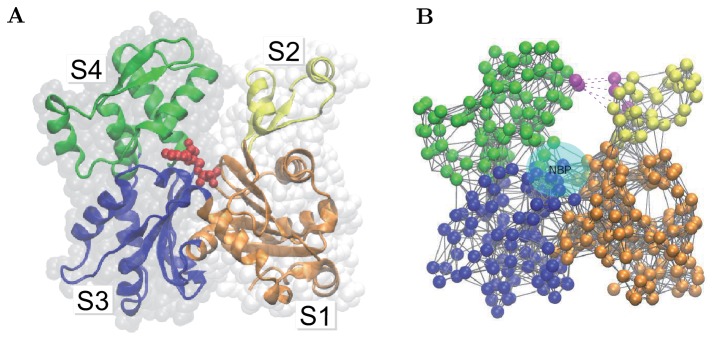
Actin and its elastic network: (A) G-actin in the ribbon representation, colored according to its subdomains S1 (orange), S2 (yellow), S3 (blue) and S4 (green). The bound ADP molecule (red) is shown. (B) The elastic network of G-actin, colored in the same way. Magenta dotted lines indicate breakable links (Lennard-Jones type bonds) between some residues (also marked magenta) in the subdomains S2 and S4. The nucleotide-binding pocket (NBP) is schematically displayed.

In this study, actin is described in terms of the coarse-grained anisotropic network model [42], [43]. In the EN approach, a protein is considered as a mechanical object formed by a network of beads connected by elastic links. The network structure is deduced from experimental data. In Fig. 1, G-actin in its ribbon representation and the corresponding elastic network are shown. Each residue is modeled as a bead and neighboring amino acids are connected by elastic links with the same stiffness if their equilibrium distance is smaller than a certain cutoff length 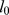. Thus, the elastic energy of the network is 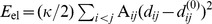. Here, 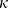 is the stiffness of the elastic links, 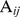 the connectivity matrix, 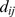 the distance between nodes and 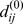 the corresponding equilibrium distance. The equations of motion describing the dynamics of the actin elastic network are formulated in the Methods section. Note that in the rescaled units used, the forces are measured in 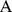, i.e. a force of 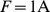 stretches a single elastic link by 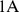.

As a reference state for the construction of the elastic network, the uncomplexed G-actin in the ADP-bound state (PDB ID: 1J6Z) was used [4]. For comparison, the elastic network of the F-actin model (PDB ID: 3MFP), obtained by fitting to cryo-electron microscopy data, was chosen [11]. The G-actin data consists of 372 residues divided into two major domains, known as the outer and the inner domains. They are separated by a cleft in which the nucleotide binds. Traditionally, each of them is further divided into two subdomains [60]. The outer domain contains subdomains S1 (residues 1–32, 70–144 and 338–372) and S2 (residues 33–69). Part of subdomain S2 is the DB loop playing an important role in inter-subunit binding. The inner domain consists of subdomains S3 (residues 145–180 and 270–337) and S4 (residues 181–269). Figure 1A shows the subdomain structure of the actin monomer.

In our simulations, positions of all residues were determined at each integration step and, therefore, complete information about conformational motions was available. This full data was used, e.g., when conformational snapshots were constructed or videos of characteristic conformational motions were generated. For concise characterization, we have additionally used a set of three order parameters followed in the simulations. Specifically, distances 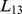 and 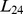 between the centers of mass of S1 and S3 and the centers of mass of S2 and S4, respectively, were chosen. As the third order parameter, the dihedral angle 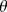, i.e. the angle between the plane defined by the mass centers of S1, S2 and S3 and the plane defined by the mass centers of S1, S3 and S4, was taken.The distance 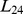 characterizes the scissor-like motion of the two mobile domains, while the angle 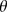 provides a characterization of the propeller-like twist (see below). The three chosen order parameters show large variation when experimentally known conformations of G- and F-actin are compared. It should be noted that these order parameters agree with the dynamical variables employed in the coarse-grained four-domain description of the actin filament by Chu and Voth [61].

### Domain motions and metastable states

In the first part of our study, characteristic global motions of actin domains, shown in Fig. 1, were investigated. Our attention was focused on large-magnitude motions for which nonlinear effects were essential and where metastable protein conformations could be approached. To systematically explore the mechanics of G-actin, randomly generated static forces were applied to all residues forming the protein and, by integrating equations (3), various deformed states were obtained. Additionally, we considered conformational relaxation processes from such deformed states to the equilibrium conformation of G-actin once the external forces had been removed.

Generally, application of a static force induces rigid translations and rotations of the entire protein. To eliminate such effects, additional balancing forces were computed at each integration step and applied to the network. They were chosen in such a way that only global translations and rotations could be caused - and thus compensated - by them, without any internal deformations arising. This immobilization procedure was the same as in Ref. [18], where its detailed description can also be found. We have always used it in the presence of external forces in our current investigations.

To generate static forces, for each residue a direction was randomly chosen and the force magnitude was randomly selected from the interval between 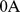 and 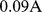. Such independently generated random forces were applied to all network nodes and a new stationary configuration of the network in the presence of the forces was determined by integrating for sufficiently long time the equations of motion (3), until a stationary state in the presence of forces was reached. Subsequently, the forces were lifted and a conformational relaxation process was followed by integrating the same equations.

**Figure 2:**
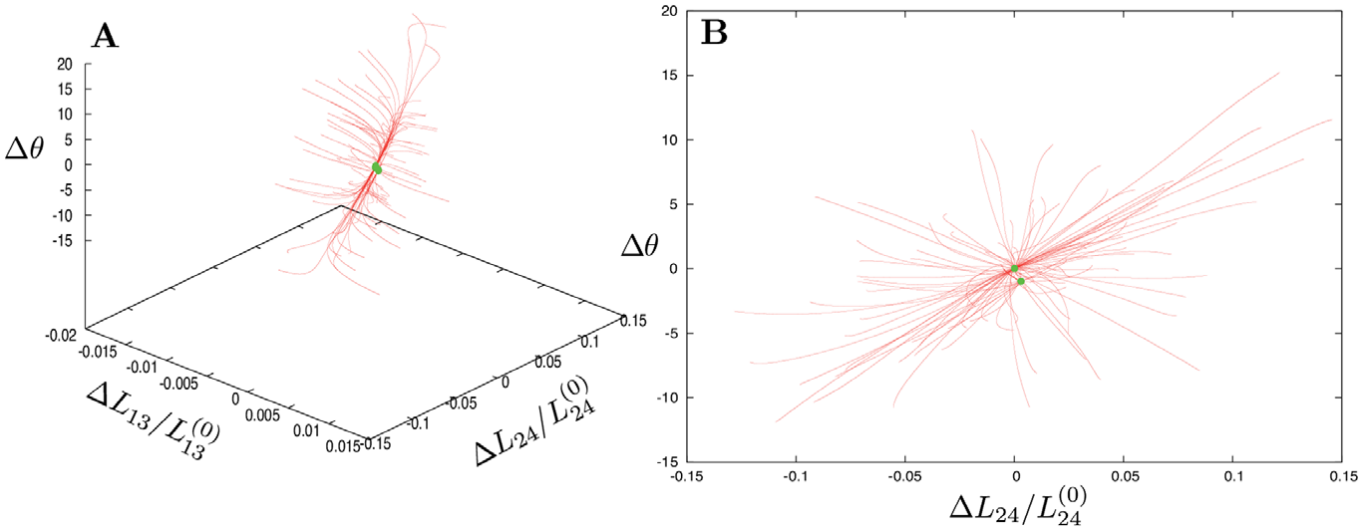
Responses to global perturbations. 100 relaxation trajectories (red curves) start from different initial conditions, generated by application of random, globally distributed static forces. Each trajectory begins from a stationary state obtained after application of a different random set of static forces. Such forces are removed when the subsequent relaxation trajectories are considered. The final states for each of these trajectories are marked by green points. Panel B shows the projection of relaxation trajectories on the plane defined by the dihedral angle and the distance between the mass centers of the two mobile subdomains.

Figure 2 displays results of such simulations for 100 different choices of random forces and, thus, for 100 different relaxation trajectories. The initial positions of the trajectories correspond to the stationary states of the network in the presence of random external forces. Hence, they characterize conformational responses of the network. As we see, the distance 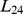 between subdomains S2 and S4 can change considerably, i.e. up to 15%, and the dihedral angle 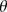 between the inner and outer domains can undergo variation up to 15 degrees. Significant changes in the distance 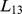 between the lower subdomains S1 and S3 were not found in our simulations. The experimentally observed difference of about 13% in the distance 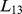 in F-actin, as compared to the equilibrium state of G-actin, can be a consequence of the interactions between monomers in the actin filament.

When external forces were switched off, the network was undergoing relaxation back to its equilibrium conformation. The relaxation trajectories starting from different initial deformed states are displayed in Fig. 2. The end points of the trajectories (green dots) correspond to the finally reached states; the farthest points of the trajectories represent the starting positions. There are only two such end points in Fig. 2. One of them lies in the origin of coordinates and thus corresponds to the equilibrium state of the elastic network. We have checked that the second state corresponds to a small buckling of a single residue within the flexible part of subdomain S4 and thus represents only a slight local modification of the equilibrium state. Even after relatively large deformations the network always returns to the equilibrium state or to its slight modification.

Thus, we see that the two principal mobile domains of actin are able to perform large-magnitude motions characterized by substantial changes of the distances between S2 and S4, as well as of the dihedral angle between the two mobile domains. The displacements of residues, accompanying such motions, are large and the linearized description and the normal-mode analysis are not justified in this case (cf. the discussion in Refs. [18] and [51]). Nonetheless, such approximate descriptions can be still employed for qualitative understanding and interpretation of the observed motions.

We have computed the normal modes of G-actin in the framework of the EN approximation used in the present study (see Supplementary Movies). The slowest normal mode of the elastic network corresponds to the propeller-like twist of the two mobile domains which can be well characterized by the dihedral angle (see Movie S1). The second slowest normal mode represents the scissor-like opening or closing of the two domains, as seen in Movie S2. These characteristic motions have been previously identified by Tirion and ben-Avraham [58] in the framework of a different normal-mode analysis where all actin atoms were resolved and only angle variations of the bond angles were taken into account. They are well reproduced in the anisotropic EN model used in our study.

Our analysis based on full nonlinear equations of the elastic network indicates that, for some large-amplitude motions, the cleft separating subdomains S2 and S4 almost disappears, so that the residues belonging to opposite domains could come near one to another. In such situations, the EN model needs to be modified, as explained below.

When an elastic network for a protein is constructed, distances between all pairs of residues in the equilibrium reference state are checked and elastic links are introduced whenever the distance between a pair is shorter than the cut-off length. Suppose now that some residues, well separated at equilibrium, come close when a perturbation is applied. If we want to follow the concept of the EN approximation, additional links connecting such residues would need to be introduced once they are close one to another. Such emergent (and breakable) links cannot be elastic, instead they should be described by a pair interaction potential which becomes flat as the distance between the particles increases. Hence, they would be effectively present only when the two particles are close one to another - and would disappear when the particles are far apart.

To allow such bonds to emerge, we add into the EN model a set of breakable links between those G-actin residues from opposite domains which are connected by elastic links in the EN model of F-actin. Thus, the original EN model of G-actin is expanded by us through the introduction of five additional breakable links connecting pairs of residues 62–204, 63–203, 63–204, 66–203 and 67–203. The new links are described by truncated Lennard-Jones potential (6) with the interaction parameters given in the Methods section, where also further details and discussion can be found.

**Figure 3:**
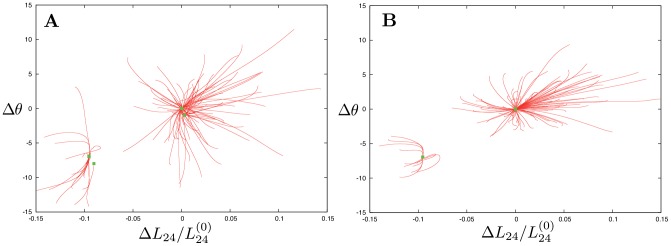
Responses to global perturbations (A) and to local pertubations of sensitive residues in the NBP region (B) are shown in the presence of breakable bonds. 100 relaxation trajectories (red curves) start from random initial conditions. The final states for each initial deformation are marked by green points. In addition to the equilibrium, metastable closed states are observed.

Taking the expanded EN model, global mechanical responses of the elastic network were examined and its relaxation trajectories were explored using the same procedure as described above for the original network. The results are displayed in Fig. 3A. Not surprisingly, the relaxation behavior remains essentially the same in the neighborhood of the equilibrium reference state of G-actin, which defines the origin of coordinates. However, an important change is observed in the region corresponding to the closed actin conformations. Previously, such conformations could be easily visited in response to mechanical perturbations, but the network was always returning from them back to the equilibrium reference state (cf. Fig. 2). In contrast, the expanded EN model of G-actin possesses a new stationary closed state, stable with respect to sufficiently small perturbations. Its origin is clear: if the two mobile subdomains are brought close enough one to another, cross links connecting them are established and, thus, the closed actin conformation becomes locked.

Actually, not one, but two closed metastable states of actin can be discerned in Fig. 3A. A detailed examination of them reveals that they differ only by local buckling in the flexible region of subdomain S4, involving a single residue, whereas the global domain configuration is the same in both of them. As can be seen in Fig. 3A, the metastable state is characterized by a closed cleft between subdomains S2 and S4 and a smaller dihedral angle 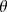, i.e. by a flattening of the molecule.

Summarizing the results of our investigations, we conclude that large-amplitude propeller and scissor motions of the principal mobile domains can take place in the elastic network of G-actin. These motions are generic and the protein responds by them when random globally distributed perturbations are applied. Moreover, we find that, in the expanded version of the EN model, the protein can also be found in the closed stationary conformation which represents its new metastable state. A transition from the equilibrium reference conformation to this metastable state can be induced by applying appropriate perturbations to the network nodes.

### Responses to perturbations in the nucleotide-binding pocket

When nucleotides (ATP or ADP) are bound to actin, this leads to local mechanical perturbations in the NBP. This pocket is located at the bottom of the cleft separating subdomains S2 and S4 (see Fig. 1). It includes a number of residues which are identified below. In the second part of our study, domain motions induced by application of static forces to individual residues in the NBP region were systematically probed.

Our attention was focused on the perturbations corresponding to the transition from the ADP- to the ATP-bound states of G-actin. The nucleotide-free state is less relevant in the context of actin polymerization and it was not analyzed here. As the reference conformation, the ADP-bound state (PDB ID: 1J6Z) was always taken. When ATP is instead bound, this means that the phosphate 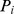 is additionally present in the pocket. Hence, only the residues in the neighborhood of phosphate should be directly affected. They are residues 12–16 in the S-loop, residues 71–75 (with the methylated histidine at the position 73) in the H-loop, residues 155–160 in the G-loop, and residue 301 in the subdomain S3.

Our analysis was performed similar to the previous investigation for myosin-V [18]. To probe the responses, static forces with randomly generated orientations and a fixed magnitude were applied to an individual residue in the chosen set, equations of motion (3) were integrated and pair distances between the subdomains, as well as the dihedral angle, were determined in the new stationary state.

To probe mechanical sensitivity of each residue, 200 simulations have been performed for each probed residue. In each of these simulations, a static force with randomly generated orientations and the magnitude 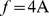 was generated and the subdomain distances 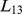, 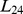 and the dihedral angle 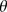 were determined in the resulting stationary state. For each residue the maximum response over the ensemble of 200 realizations was taken to characterize the sensitivity of this particular residue with respect to a certain distance or the dihedral angle.

**Figure 4:**
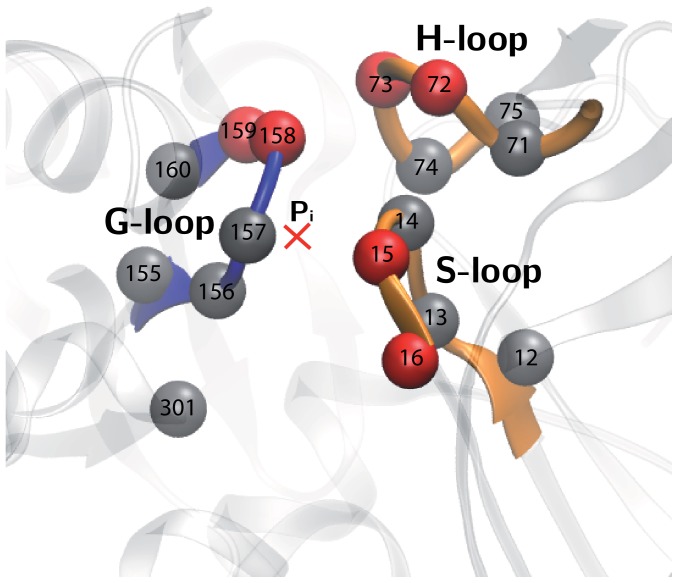
Residues in the neighborhood of the phosphate 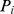 (red cross) which belong to the three sensory loops G, H and S inside the NBP. Red beads indicate the sensitive residues as identified in Table 1.

The results of the sensitivity analysis are presented in Table 1. As we see, the maximal induced changes of the distance 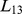 between subdomains S1 and S3 were always small and not essential. In contrast, both the distance 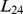 and the dihedral angle 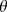 could change substantially when perturbations to certain residues were applied. According to Table 1, the sensitive residues are 15, 16, 72, 73, 158 and 159. Applying forces of magnitude 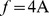 to such residues, dihedral angle changes of more than 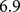 degrees and relative domain distance 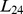 changes of more than 11% could be induced. The sensitive residues are additionally displayed in Fig. 4. Note that pairs of sensitive residues are located within each of the three important loops S, H and G.

**Table 1:** Sensitivity of selected residues in the NBP region.

Residue ID	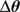	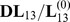	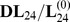
12	4.3	0.012	0.09
13	5.2	0.017	0.10
14	4.3	0.018	0.11
**15**	**8.0**	**0.019**	**0.15**
**16**	**10.0**	**0.016**	**0.15**
71	5.1	0.016	0.08
**72**	**7.8**	**0.021**	**0.13**
**73**	**7.9**	**0.026**	**0.14**
74	2.3	0.020	0.10
75	4.6	0.027	0.09
155	5.2	0.014	0.08
156	3.8	0.016	0.09
157	3.8	0.017	0.12
**158**	**6.9**	**0.020**	**0.13**
**159**	**8.4**	**0.018**	**0.11**
160	6.7	0.018	0.10
301	5.9	0.014	0.05

In the above sensitivity analysis, the original EN model was employed. As we have shown in the previous section, this model can be, however, expanded by including a set of breakable links which become effective when subdomains S2 and S4 come close one to another. Domain motion responses to the application of forces to sensitive residues in the NBP region have been further analyzed in the framework of the expanded EN model.

For the detailed analysis, only one sensitive residue in each of the three loops was chosen. Similar behavior could be expected if its neighbor in the same loop was instead selected. Thus, we focused on the responses induced by application of perturbations to the group of three residues: 16 (in G-loop), 73 (in H-loop) and 159 (in G-loop). Static forces were applied, at the same time, to all three residues in the group. The magnitude of each force was randomly chosen between 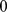 and 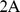 and its orientation was random. For every choice of forces, evolution equations (3) for the expanded EN model were integrated until a stationary state was reached. After that, the forces were lifted and the relaxation process was followed by integrating the same equations. The results are displayed for 100 different random perturbations in Fig. 3B.

Comparing Figs. 3A and 3B, it can be noticed that, although the forces were applied to only three NBP residues, essentially the same domain responses as for the application of globally distributed perturbations could be produced. The minor metastable states in Fig. 2, corresponding to single-residue buckling in the highly flexible region of subdomain S4 were absent because forces in that region were not applied. As we see, perturbations of the three sensitive residues already led to characteristic propeller- and scissor-like motions of the inner and outer domains. Furthermore, such local perturbations were sufficient to induce a transition to the metastable closed state of G-actin.

Thus, a small number of sensitive residues lying in the NBP region and belonging to three different loops could be identified. Applying appropriate static perturbations to a group of three such residues, each from a different group, a transition from the open to the closed state of G-actin could be reproduced. Remarkably, local deformations in the NBP region were able to spread over the elastic network and become transformed into large-amplitude global motions of mobile domains.

### Ligand-induced conformational changes

Since EN models are coarse-grained and entire residues are replaced by point-like particles, the detailed atomic structure of ligands (ATP or ADP) cannot be resolved in this approach. In this section, the ligands will be treated as additional particles. As it turns out, even this greatly simplified phenomenological description allows us to understand some important aspects of ligand-induced conformational changes.

**Figure 5:**
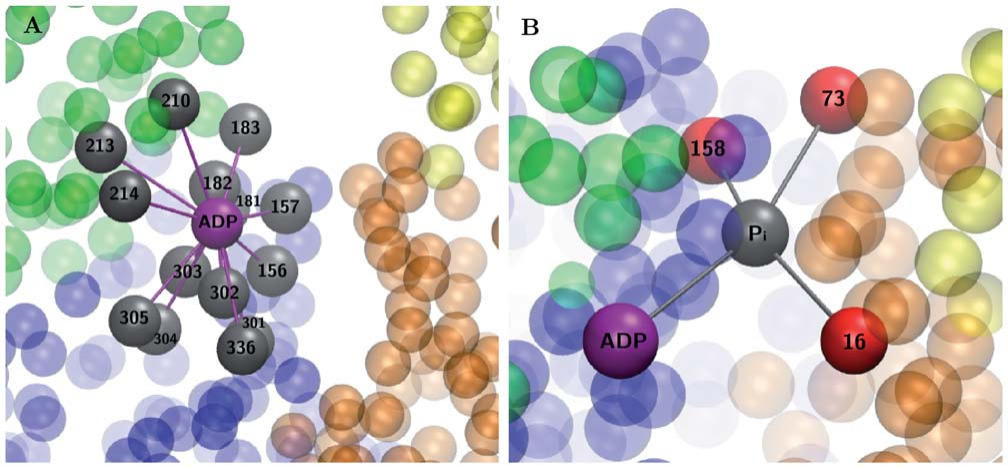
Simple modeling of ligands. (A) The ADP is modeled as an additional node (purple bead) added to the elastic network. It is connected to all its neighbors (grey beads) by elastic links. (B) The ATP is modeled as a dimer consisting of ADP (purple bead) and 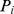 (grey bead), connected by an elastic link. The ADP is elastically connected to its neighbors and the phosphate is elastically linked to the three sensitive nodes (red beads).

**Figure 6:**
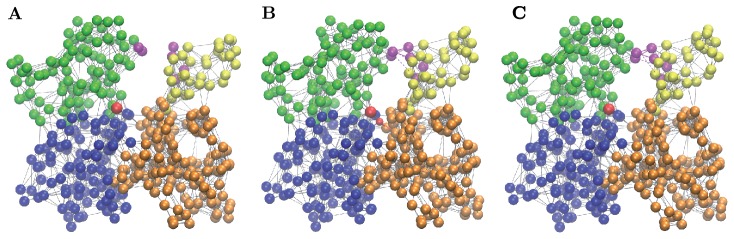
Ligand-dependent conformational states of G-actin. (A) Equilibrium state with the ADP ligand bound. (B) Equilibrium state with the ATP ligand bound. (C) Metastable state with the ADP ligand bound. ADP is shown as a bigger red bead, and 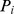 is visualized as a small red bead; ATP is modeled as a dimer consisting of ADP and Pi. Magenta-colored beads indicate residues between which additional breakable links can become established. Such breakable links are shown by solid magenta lines, if they are actually present, and by dashed lines if they are broken.

The structure of G-actin with ADP is experimentally known and it was already used by us to construct its elastic network. Below, ADP is explicitly included into the EN description. We treat it as a single particle and put this particle into the equilibrium 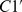 position, connecting it by elastic links to all residues within the cutoff distance 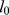 (see Fig. 5A). The natural lengths of the links are chosen equal to the equilibrium distances between 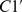 and the respective residues. Hence, by construction, the introduction of such a particle does not change the equilibrium conformation of the protein network. Because the particle is only connected to one of the subdomains (i.e. to S1), its introduction does not also significantly affect the dynamics of the mobile domains. The equilibrium state of the elastic-network of G-actin with the additional particle, modeling ADP, is shown in Fig. 6A.

When an ATP molecule is bound to actin, we model it as a dimer consisting of two particles (Fig. 5B). The first of them corresponds to the ADP part of ATP and the second of them imitates the 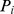. The first particle is at the same position where ADP was located in the equilibrium conformation of G-actin. The second particle is placed in the center of mass of the residues 16, 73 and 159, and the ADP. It is connected by elastic links to these four particles (see Methods for the detailed description).

In contrast to ADP, residing entirely on one of the mobile domains, the phosphate interacts with the residues from different mobile domains (cf. Fig. 4) and, thus, its arrival may induce relative domain motions. Both the X-ray diffraction experiments [4] and MD simulations [27] reveal that, in the presence of ATP, the nucleotide binding pocket becomes contracted. To approximately account for this effect, we assume that the natural lengths of the elastic links, which connect the 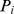 ligand particle to its neighbors, are shorter than the distances between them and the 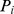 ligand when it is introduced. Namely, the natural lengths of the elastic links, connecting 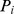 to residues 16, 73 and 159 and ADP, are chosen to be equal to 20% of the distances between these residues and the 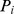 position (i.e., the center of mass of these three residues) in the reference state which corresponds to the equilibrium conformation of G-actin with ADP bound. Thus, these links are initially stretched; they tend to contract the nucleotide-binding region.

Binding of the ATP, imitated in our simple phenomenological model through the introduction of an additional 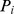 ligand, leads to a shrinking of the NBP which translates into conformational motions of the inner and outer domains. The two domains approach one another, so that within the expanded EN model the additional links connecting them become effectively established and they lock the closed conformation of the protein. This process is illustrated in the first part of the supplementary Movie S3. The final closed conformation of G-actin, stabilized by binding of ATP, is displayed in Fig. 6B.

The hydrolysis reaction and the release of phosphate are roughly imitated in our model by cutting all links which connect the 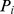 ligand to its three neighbors and the ADP and by removing this particle from the pocket. When this takes place, the actin is in its closed conformation shown in Fig. 6B. The removal of 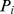 changes the interactions within the NBP and, as we observe in our numerical simulations (the second part of Movie S3), leads to a certain opening of the cleft between the two mobile domains. However, in absence of thermal fluctuations (see below), the additional links between the two domains then do not break and, after the phosphate release, the actin is found in its metastable closed state (Fig. 6C).

**Figure 7:**
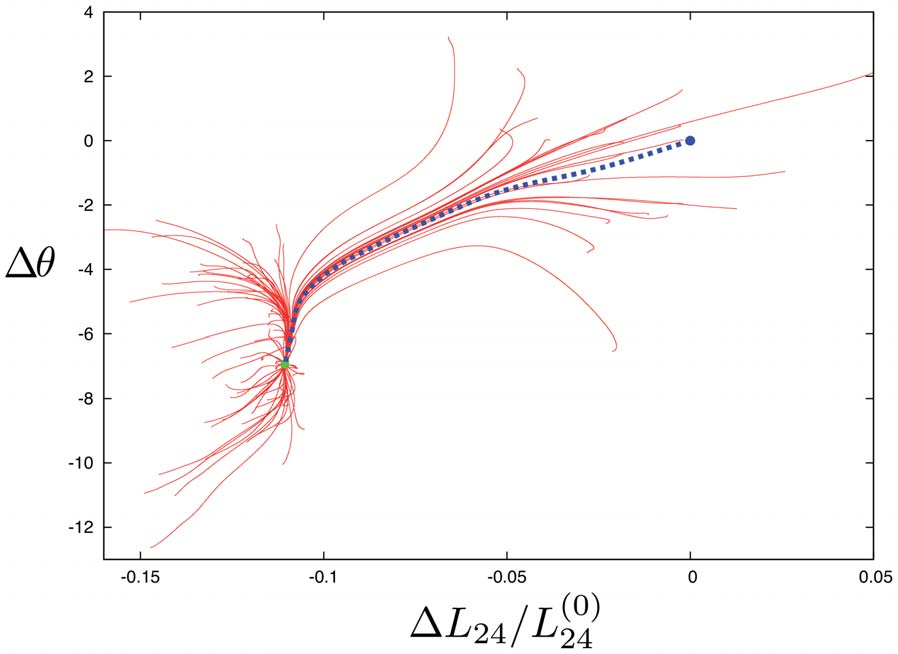
The pattern of relaxation trajectories for the ligand-network complex. The blue trajectory shows relaxation starting from the open equilibrium conformation of G-actin without the ligand. The others start from the perturbed conformations which were obtained by applying random static forces to the three sensitive residues in the NBP region. The open conformation does not correspond to a stationary state of the complex and all trajectories converge to the new equilibrium closed state indicated by the green dot.

Binding of the artificial ligand leads to a new, unique equilibrium position. 100 relaxation trajectories in the presence of the ATP ligand are shown in Fig. 7. Initial deformations were prepared by applying static external forces with random directions and an amplitude drawn from the interval [0,2 Å] to the three sensitive residues in the NBP. Starting from the equilibrium conformation of G-actin, the equations of motions (3) were integrated in the presence of the ligand until a stationary state was reached. Additionally, Fig. 7 shows the relaxation trajectory which starts from the original equilibrium state of G-actin in the absence of a ligand. As revealed by Fig. 7, binding of the ligand makes the open conformation unstable and stabilizes the closed actin conformation.

So far, effects of thermal fluctuations have been excluded from our analysis. Such effects may become, however, important if metastable states are possible. If thermal fluctuations are strong enough, they can induce transitions between stable and metastable states, so that all of them can be visited by the system.

**Figure 8:**
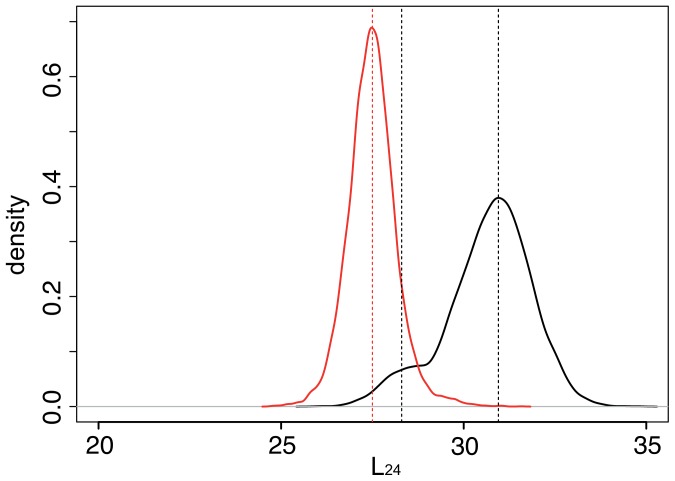
Statistical distributions of interdomain distances 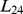 in G-actin under thermal noise in the presence of ADP (black) or ATP (red) ligands.

The effects of thermal fluctuations can be taken into account by introducing additional random forces with appropriate intensities into the equations of motions (see Methods). Integrating such stochastic differential equations over sufficiently long time, data was gathered and statistical distributions for various order parameters in the presence of different ligands (ADP or ATP) were constructed. Figure 8 displays statistical distributions of the distance 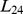 between the centers of mass of the mobile domains S2 and S4 in the ADP- or ATP-bound states, as described by our approximate model. In the ADP-bound state (black curve), the protein prefers to stay in the open conformation, with the distance between the domains approximately equal to 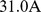. The closed conformation, representing a metastable state, is however also occasionally visited, as evidenced by the presence of a shoulder in the statistical distribution of the interdomain distances. Binding of ATP stabilizes the closed conformation, leading to the distance distribution shown by the red curve in Fig. 8. In the presence of ATP, spontaneous transitions to the open conformation are not possible (or very rare), as evidenced by the presence of a clear distribution maximum at the distance 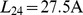 in this case. The width of the distance distributions characterizes the stiffness of the monomer. With ATP bound, the variance of the distance 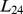 is reduced to 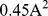, as compared to the variance of 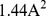 in the ADP-bound state. Thus, the presence of ATP in the NBP stiffens the monomer considerably.

Already the rough modeling employed in this section reveals some important effects of the nucleotides. Binding of ATP can directly lead to flattening of the protein and closing of the cleft between its inner and outer domains. While the ATP-free actin shows the tendency to switch between its two equilibrium states, the ligand can stabilize the closed conformation of actin and, furthermore, stiffen the macromolecule. Note that the structural details of the ligand-induced closed conformational state may depend on the parameters of interactions between the ligand particle and the NBP residues. Moreover, the dimer model of ATP used in the above simulations represents only a simple approximation for the actual ATP molecule. Therefore, the results of our numerical investigations including the ligand should be viewed as only providing a demonstration that a transition to a stable closed conformation can be induced by ATP binding. This prediction can be further tested by experiments and special MD simulations.

## Discussion

In our study, the attention was focused on purely mechanical aspects of actin dynamics. With this purpose, a greatly simplified dynamical model of this molecule was considered where all residues, independent of their chemical differences, were treated as identical particles connected by identical elastic links. In addition to the elastic links, the mechanical model also included a small number of breakable links that become established when pairs of residues come sufficiently close and break down at large separations. The information about the chemical structure of G-actin was effectively encoded only in the architecture of the elastic network, determined by the experimentally known equilibrium conformation of the molecule.

Remarkably, this greatly simplified model has allowed us to understand various aspects of intramolecular conformational motions in actin monomers. The model shows that the mobile inner and outer domains of actin are able to perform large-amplitude propeller twist and scissor-like motions, earlier revealed by the normal-mode analysis for small deviations from the equilibrium state [58]. While performing such motions, two upper subdomains (S2 and S4) can come so close one to another that attractive interactions between pairs of residues from the opposite domains become present. Such emergent interactions can lock the actin molecule in its closed conformation and thus lead to the formation of a metastable state.

We have found that, similar to myosin [16]–[18], [62], G-actin essentially behaves as a strain sensor, responding by well-defined domain motions to mechanical perturbations. In our previous study [18], we could identify a number of sensitive residues in the front- and back-door regions within the NBP of myosin-V, such that small perturbations of these residues were translated into large-amplitude motions of the tail and into the closing or opening of the actin binding cleft. Our present investigations of actin reveal three pairs of sensitive residues, belonging to different domain loops inside the NBP. Application of small perturbations to these particular residues can result in large-amplitude domain motions and in the transition to the metastable closed state. As we see, the internal mechanics of an actin macromolecule is highly organized and efficient communication between the NBP region and the mobile domains is present.

To demonstrate that ligand (i.e. ATP) binding can indeed induce large-scale conformational changes, we have imitated the ligand by a dimer; one of the particles, corresponding to phosphate, has attractive interactions with the sensitive residues inside the NBP. Previously, a similar ligand description was employed when cyclic operation of the molecular motor hepatitis C virus helicase was analyzed [52]. We have found that, under an appropriate choice of the interaction parameters, binding of ATP can induce a transition to the closed conformation and stabilize this metastable state.

In the hierarchy of coarse-grained models proposed to describe actin monomers and filaments (see [61] and the review [63]), the employed description is most closely resolving the structure of the individual proteins. Nonetheless, because of the simplifications involved in the formulation of the EN model and since some of the parameters, particularly referring to the interactions with ligands, remained arbitrary in the present study, quantitative agreement between the predictions based on the present coarse-grained description and the experimental data or the data of all-atom MD simulations should not be expected. The results of our approximate analysis, however, can be used for better understanding of intramolecular dynamics of G-actin. They may provide helpful guidelines for further experimental investigations and act as motivation for MD studies.

The ATP-induced transition to the closed conformation of actin can play an important role in the explanation why, in the presence of ATP, the growth of actin filaments is strongly accelerated. The closed conformation of G-actin, stabilized under ATP binding, is not identical to that of the filamentous F-actin. However, in both of these conformations the cleft separating the upper mobile subdomains S2 and S4 is strongly reduced, so that a better fit and higher affinity to the actin filament may result. Another effect of binding of ATP, observed in our model, is the increased stiffness as compared to the ADP state (see Fig. 8). This is in agreement with the experimental data showing that the presence of ATP-bound protomers leads to an increased stiffness of the filaments [64].

In recent experiments [12], metastable conformational states of single actin protomers in the filament could be already detected. The distribution of these states was sensitive to addition of myosin. Actin binding proteins (ABPs), including myosin, play crucial roles in the cell [65]. In the framework of our approach, interactions with ABPs can be interpreted as mechanical perturbations and can also be analyzed in future studies. It should also be possible to perform FRET measurements in single G-actin molecules under controlled conditions, thus elucidating conformational states involved in polymerization (G-F transitions), and specifically, the effects of ligands. The results of such experiments can be compared with the predictions based on the elastic-network models.

## Methods

### The Elastic Network Model

In the present study, conformational dynamics in actin monomers, known as G-actin, is investigated in the framework of the anisotropic EN model [42], [43]. In this EN model, an elastic network is formed by 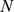 identical point particles (nodes) which are connected by identical elastic links. The network architecture is defined by the experimentally known equilibrium positions 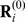 of all residues 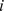 in the protein. For this purpose, the positions of 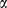-carbon atoms are taken. If the equilibrium distance 
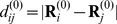
 between two nodes 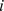 and 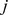 is smaller than some cutoff 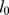, a link is introduced. The natural length of the link is chosen equal to the respective equilibrium distance 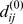. Note that, by construction, the equilibrium conformation represents the state with the lowest elastic energy. Fig. 1 displays equilibrium structures of G- actin together with the elastic network of this protein.

The total elastic energy of the network is given by the sum of the energy stored in its elastic links
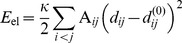
(1)where 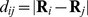 is the distance between two particles 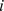 and 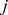 and the elements of the 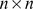 adjacency matrix 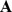 are 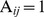, if 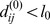, and 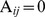 otherwise. The spring stiffness constant 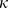 is the same for all nodes. If external forces 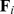 are applied to the network, its energy is 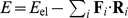.

In our study, the cutoff length of G-actin in the anisotropic EN has been determined by following the arguments by Atilgan et al. [66]. A sequence of EN models with gradually increasing values of the cutoff length was constructed and, for each cutoff length, the eigenvalues of the linearization matrix (see below) were computed. If the cutoff length was too small, the network was falling into disconnected components or free rotations inside it were possible, as evidenced by the fact that more than six zero eigenvalues of the linearization matrix were found. The cutoff length of 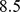 Å, used in our numerical investigations, was chosen as the first cutoff length at which only six zero eigenvalues were present. Note that, in the study [66], a slightly higher cutoff length of 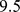 Å was found to hold in the anisotropic network approximation for a large group of proteins (but actin was not considered there).

On the considered time scale of milliseconds, inertial effects are negligible and conformational dynamics is purely dissipative [67]. In our present study, hydrodynamical interactions between particles will be neglected. In the overdamped limit, particle velocities are proportional to the forces acting on it and the equations of motion are
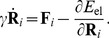
(2)We assume that the friction coefficient 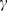 is equal for all particles. After an appropriate rescaling of time, the parameters 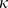 and 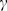 can be removed from these equations and they take the form
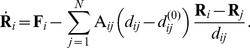
(3)Note that, in the new units employed, the force 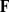 is measured in Å. A force of 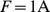 is stretching a single elastic link by 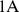. An important property of equations (3) is that they are generally nonlinear in terms of the coordinates 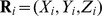 because of the nonlinear dependence of the distances 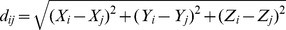 on these variables.

The dynamics of the EN is followed by integrating equations (3) using the explicit Euler method with the time step 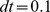. By repeating integrations for some relaxation trajectories with a smaller time step of 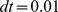, we have checked that this does not lead to significant changes. To check this, we have compared the two stationary states in the presence of external forces that were obtained by integrating the equations of motion (3) with two different time steps of 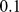 and 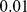. The time evolution of the sum of distances between all residues in the network were followed in both simulations and the differences in distance between the corresponding nodes were found to be below 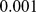 Å. Thus, the decrease of the time step did not lead to an accuracy improvement and the choice of the time step as 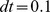 was sufficient for our analysis.

Integration of relaxation trajectories has been continued until a stationary state was reached. The numerical criterion was that the sum of the distances between the centers of mass of four actin subdomains and the dihedral angle have ceased to change by more than 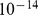 Å and 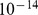 degrees per step, respectively.

Generally, application of external forces can induce rigid rotations and translations of the entire elastic object. To eliminate such global motions, some immobilization procedure needs to be employed. The easiest immobilization solution would have been to pin three network particles. However, the disadvantage of such simple method is that, depending on the choice of the pinned particles, various internal deformations of the network may arise. As an alternative, we have previously proposed a computational immobilization method which does not lead itself to network deformations [18]. In this method, additional forces that balance out rigid rotations and translations are computed at each integration step and applied to all network particles. The detailed description of this method, which was also used in the present study, can be found in Ref. [18]. In our present study, immobilization was always used if external static forces were applied to the EN.

### Linearized Equations of Motion

The equations of motions (3) of the EN model are nonlinear because distances are nonlinear functions of the coordinates. EN models are often used for the normal-mode analysis [39]–[47]. Then, only small displacements of particles from their equilibrum positions are considered. The linearized equations are
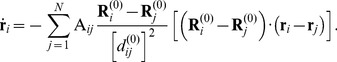
(4)They hold for small deviations 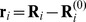 from the equilibrium. Equations (4) can also be written in the matrix form 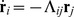, with a 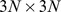 matrix 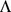. The linearized equations of motion can be solved analytically yielding the normal relaxation modes. In terms of the eigenvalues 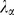 and the eigenvectors 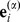, the general solution is
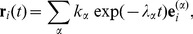
(5)where coefficients 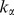 are determined by the initial conditions. Thus, the eigenvalues determine the relaxation rate constants of the respective normal modes. The slowest relaxation processes are controlled by the normal modes with the lowest eigenvalues.

Assuming that only one of the normal modes is excited, the motions of network particles are given by equations (5) where only one term, corresponding to a particular mode, is present. Thus, characteristic network motions in a specific normal mode can be determined and visualized.

The two slowest normal modes for the elastic network of G-actin have been computed and the respective motions are displayed in Movies S1 and S2. The slowest normal mode (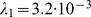) corresponds to the scissor-like motion of the inner and outer domains (Movie S1). The second slowest mode (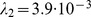) produces the propeller-like twist of the two domains with respect to each other.

The linearized equations (4) and, thus, the normal-mode description are justified only while the displacements 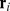 remain much smaller than the equilibrium distances between the particles connected by the elastic bonds [18], [51]. Since such distances, by construction, cannot exceed the cutoff length, the displacements must be much smaller than 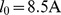. This condition is not satisfied for the motions approaching the closed conformation of G-actin, which should be therefore treated in the framework of the full EN description. Nonetheless, the slowest normal modes are still in qualitative agreement with the results of the nonlinear simulations.

The equations of motion (3) depend only on relative distance changes. Therefore, they are always invariant against rigid translations and rotations of the entire elastic network. This implies that the linearization matrix 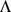 should always have six zero eigenvalues. On the other hand, if more than six zero eigenvalues are found, this indicates that the network breaks down into disconnected components or that free internal rotations of some residue groups are possible. This property can be used in the selection of the cutoff length, as explained above.

### Breakable Links

Studying the response to external forces, we notice that the distance 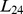 between the centers of mass of subdomains S2 and S4 can decrease considerably (see Fig. 2), i.e. residues in these subdomains can come very close to each other. Within the EN description, however, links should be present if residues are within the cutoff distance 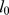. Thus, a set of several breakable links has additionally been introduced into the model. Pairs of interacting residues have been identified by using the structural data for the F-actin (PDB ID: 3MFP) conformation. For simplicity, we have assumed that only the five additional pairs of residues closest to each other in this conformation establish breakable bonds. These pairs of residues are 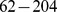, 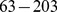, 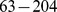, 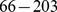, and 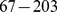 (such residues are outlined in Fig. 1B).

In contrast to regular elastic links, breakable links are described by the truncated Lennard-Jones potential
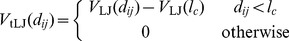
(6)with the function
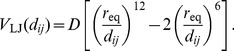
(7)The interaction parameters are 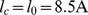, 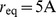 and 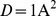. These links are effective only when the distances between the two residues are below the truncation length 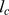.

When breakable bonds are added, equations of motion (3) should be modified by including additional forces
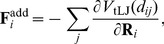
(8)where the summation is performed over the subset of nodes 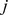 which are connected by additional breakable links to the node 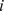.

Our choice of the interaction parameters for breakable links was based on the requirements that (i) this potential becomes flat when distances between the residues exceed the cutoff length of 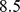 Å used in the construction of the elastic network from the experimental data, (ii) the minimum of the interaction potential is found at the distance which lies between the minimum and the maximum values of the natural lengths 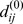 of elastic links, as deduced from the experimental data for G-actin, and (iii) when breakable links are effective, they lead to attraction forces between the domains which are similar to the forces which would have been generated by regular elastic links if they were instead present.

Comparing the experimentally known conformations of the globular G-actin and the filamentous F-actin [4], [11], one can notice that, under the cutoff length of 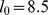 Å, there are eleven additional links between the subdomains S2 and S4 in the F-actin structure. Generally, breakable links may be introduced between all such eleven residue pairs. We have selected, however, only the five closest pairs of residues and introduced breakable links between them. We have chosen the same value of the equilibrium distance 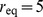 Å for all additional links. With the choice of 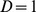 Å^2^ the interaction potential (7) near the equilibrium distance was by a factor of 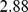 stronger than that of the regular elastic links. By choosing 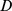 in this way, we could approximately compensate for the smaller number of interacting pairs in our model as compared to the experimental F-actin structure. Moreover, this could take approximately into account that the potential (7) becomes flat whereas the elastic forces continue to increase as the cutoff distance is approached.

We have also repeated some of the simulations using soft breakable links with 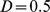 Å^2^. Supporting Figure S1 shows patterns of relaxation trajectories in the absence or presence of the ATP ligand in this case. They can be compared with the respective Fig. 3B and Fig. 7 in the main text. When the ligand is absent (Fig. S1A), a metastable stationary state is not found, but the respective closed conformation can still be easily visited as a result of perturbations. Binding of the ligand makes the open conformation unstable and creates a stable state corresponding to the closed conformation of the protein (Fig. S1B). Thus, ATP-induced stabilization of the closed conformation of G-actin persists if soft breakable links are chosen. In contrast, the metastable closed conformation in the absence of the ligand is found only if breakable links are strong enough.

### Residues near 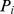 in the NBP

An important actin binding protein is the bovine pancreatic deoxyribonuclease (DNase I). Complexed with DNase I, a structure of the actin monomer with ATP bound (PDB ID: 1ATN) was experimentally resolved [60]. Accordingly, the position of the phosphate is known for this structure. Using this information, residues in the NBP that can interact with the 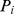 can be specified. Within a distance of 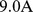 from the phosphate position, residues 12–16 in the S-loop, residues 71–75 (with the methylated histidine at the position 73) in the H-loop, residues 155–160 in the G-loop, and residue 301 in the subdomain S3 are found. This set of residues is shown in Fig. 4 and has been selected to probe the mechanical responses.

### Ligand Modeling

In our numerical investigations, a greatly simplified phenomenological description of ligands ADP and ATP as a point particle and a dimer has been employed. ADP was modeled by introducing an additional node in the network whose equilibrium coordinates were chosen to coincide with the position of the 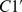 atom. This carbon atom connected the nucleotide's ribose with the adenine and, therefore, was located in the center of the ADP molecule. The new node was connected to all neighboring residues within the cutoff distance 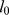 by elastic links of stiffness 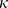, the same as for the elastic network of the protein. The neighbors of ADP were residues 156, 157, 181–181, 301–305, and 336 in subdomain S3 and residues 210, 213, and 214 in subdomain S4 (Fig. 5A). The natural lengths of all elastic links were chosen equal to the respective distances in the equilibrium conformation.

When ATP was bound, it was modeled as a dimer which consists of two particles, ADP and 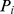. The phosphate was modeled as a node which is linked to the ADP node and three sensitive key residues 16, 73, 159. It was placed in the center of mass of these four nodes and connected by elastic links, again with the same stiffness 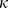 as for the protein network (see Fig. 5B). It is known that, when ATP is bound, this leads to shrinking of the nucleotide-binding pocket [27]. To account for this, we assumed in our model that the natural lengths of the links between 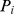 and its neighbors were by 80% shorter than the distances between the position of the 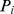 node and four other nodes in the equilibrium conformation of ADP-bound actin.

### Thermal Fluctuations

In the final part of our study, some effects of thermal fluctuations are considered. Neglecting hydrodynamic effects, this is done by introducing appropriate random forces into the dynamical equations, i.e. by writing them as
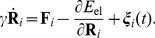
(9)Here, 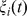 is Gaussian noise with the correlations
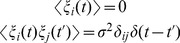
(10)in each direction separately. The parameter 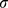 specifies the noise intensity. It is related to the temperature 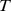 as 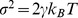. In our simulations, the value 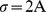 has been chosen. We have checked that, with this choice of the parameter, the experimentally known B-factors are reproduced by the order of magnitude. The simulations including thermal noise are intended to provide only an illustration of the fluctuation effects and, therefore, we have not attempted to obtain a better fitting.

Our simulations with thermal noise are used to obtain a statistical distribution of the interdomain distances 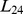. Distances are sampled every 400 integration steps and 10000 sample points have been used to obtain the distribution in Fig. 8. The first 20000 integration steps were discarded before sampling to eliminate a possible effect of initial conditions.

## Supporting Information

Figure S1
**Patterns of relaxation trajectories for the elastic network of G-actin with the soft breakable links of strength** 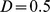 **Å^2^ in absence (A) and in the presence (B) of the ATP ligand.** 100 relaxation trajectories starting from the initial conditions obtained by application of random static external forces to three sensitive residues in the NBP region are shown. The orientations of forces are random and their amplitudes are drawn at random from the interval between 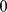 and 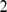 Å. The blue curve shows the relaxation trajectory starting from the open equilibrium conformation of G-actin. As observed in panel A, the metastable state is absent when soft breakabale links are chosen. Nonetheless, the respective open protein conformations are easily visited as a result of perturbations. According to panel B, ligand binding leads to the appearance of a stable closed conformational state, whereas the open state of the protein becomes unstable.(TIFF)

Movie S1 **Visualization of the motion corresponding to the lowest eigenmode of the EN model of G-actin: the propeller-like twist of outer and inner domains with respect to each other.**
(MOV)

Movie S2 **Visualization of the motion corresponding to the second lowest eigenmode of the EN model of actin: the scissor-like opening and closing of the cleft between subdomains S2 and S4.**
(MOV)

Movie S3
**Ligand-induced conformational motions.** The initial state is the equilibrium conformation of G-actin with ADP bound. Introducing an additional network node which corresponds to 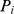, a transition to the closed state is observed. When this state is reached, the 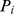 node is removed. As can be seen, the elastic network does not return then to the initial equilibrium state of ADP-bound G-actin. Instead, the actin remains in a metastable closed conformation.(MOV)

## References

[1] Alberts B, Johnson A, Lewis J, Raff M, Roberts K, et al.. (2008) Molecular Biology of the Cell. Garland Press, 5th edition.

[2] Pollard TD (1986) Rate constants for the reactions of ATP- and ADP-actin with the ends of actin filaments. The Journal of Cell Biology 103: 2747–54. 3793756 10.1083/jcb.103.6.2747 PMC2114620

[3] Korn ED, Carlier MF, Pantaloni D (1987) Actin polymerization and ATP hydrolysis. Science 238: 638–644. 3672117 10.1126/science.3672117

[4] Otterbein LR, Graceffa P, Dominguez R (2001) The crystal structure of uncomplexed actin in the ADP state. Science 293: 708–11. 11474115 10.1126/science.1059700

[5] Graceffa P, Dominguez R (2003) Crystal structure of monomeric actin in the ATP state. Structural basis of nucleotide-dependent actin dynamics. The Journal of Biological Chemistry 278: 34172–80. 12813032 10.1074/jbc.M303689200

[6] Wang H, Robinson RC, Burtnick LD (2010) The structure of native G-actin. Cytoskeleton 67: 456–465. 20540085 10.1002/cm.20458

[7] Holmes KC, Popp D, Gebhard W, Kabsch W (1990) Atomic model of the actin filament. Nature 347: 44–49. 2395461 10.1038/347044a0

[8] Holmes K, Angert I, Kull F, Jahn W (2003) Electron cryo-microscopy shows how strong binding of myosin to actin releases nucleotide. Nature 425: 423–427. 14508495 10.1038/nature02005

[9] Splettstoesser T, Holmes KC, Noé F, Smith JC (2011) Structural modeling and molecular dynamics simulation of the actin filament. Proteins 79: 2033–43. 21557314 10.1002/prot.23017

[10] Oda T, Iwasa M, Aihara T, Maéda Y, Narita A (2009) The nature of the globular- to fibrous-actin transition. Nature 457: 441–5. 19158791 10.1038/nature07685

[11] Fujii T, Iwane AH, Yanagida T, Namba K (2010) Direct visualization of secondary structures of F-actin by electron cryomicroscopy. Nature 467: 724–728. 20844487 10.1038/nature09372

[12] Kozuka J, Yokota H, Arai Y, Ishii Y, Yanagida T (2006) Dynamic polymorphism of single actin molecules in the actin filament. Nature Chemical Biology 2: 83–6. 16415860 10.1038/nchembio763

[13] Hanson JA, Duderstadt K, Watkins LP, Bhattacharyya S, Brokaw J, et al (2007) Illuminating the mechanistic roles of enzyme conformational dynamics. Proceedings of the National Academy of Sciences of the United States of America 104: 18055–18060. 17989222 10.1073/pnas.0708600104 PMC2084295

[14] Korkut A, Hendrickson WA (2009) Computation of conformational transitions in proteins by virtual atom molecular mechanics as validated in application to adenylate kinase. Proceedings of the National Academy of Sciences of the United States of America 106: 15673–15678. 19706894 10.1073/pnas.0907684106 PMC2731797

[15] Santoso Y, Joyce CM, Potapova O, Le Reste L, Hohlbein J, et al (2010) Conformational transitions in DNA polymerase I revealed by single-molecule FRET. Proceedings of the National Academy of Sciences of the United States of America 107: 715–720. 20080740 10.1073/pnas.0910909107 PMC2818957

[16] Iwaki M, Iwane AH, Shimokawa T, Cooke R, Yanagida T (2009) Brownian search-and-catch mechanism for myosin-VI steps. Nature Chemical Biology 5: 403–405. 19430485 10.1038/nchembio.171

[17] Oguchi Y, Mikhailenko SV, Ohki T, Olivares AO, De La Cruz EM, et al (2010) Robust processivity of myosin V under off-axis loads. Nature Chemical Biology 6: 300–305. 20228794 10.1038/nchembio.322 PMC2917589

[18] Dffuttmann M, Togashi Y, Yanagida T, Mikhailov AS (2012) Myosin-V as a Mechanical Sensor : An Elastic Network Study. Biophysical Journal 102: 542–551. 22325277 10.1016/j.bpj.2011.12.013 PMC3274815

[19] Izrailev S, Stepaniants S, Balsera M, Oono Y, Schulten K (1997) Molecular dynamics study of unbinding of the avidin-biotin complex. Biophysical Journal 72: 1568–1581. 9083662 10.1016/S0006-3495(97)78804-0 PMC1184352

[20] Sugita Y, Okamoto Y (1999) Replica-exchange molecular dynamics method for protein folding. Chemical Physics Letters 314: 141–151.

[21] Cecchini M, Alexeev Y, Karplus M (2010) Pi release from myosin: a simulation analysis of possible pathways. Structure 18: 458–70. 20399183 10.1016/j.str.2010.01.014 PMC2858069

[22] Elber R (1990) Calculation of the potential of mean force using molecular dynamics with linear constraints: An application to a conformational transition in a solvated dipeptide. The Journal of Chemical Physics 93: 4312–4321.

[23] Fine R, Dimmler G, Levinthal C (1991) FASTRUN: a special purpose, hardwired computer for molecular simulation. Proteins 11: 242–253. 1758880 10.1002/prot.340110403

[24] Narumi T, Taiji M, Ikei M, Ohno Y, Okimoto N, et al (2006) A 55 TFLOPS simulation of amyloidforming peptides from yeast prion Sup35 with the special-purpose computer system MDGRAPE-3. Proceedings of the 2006 ACM/IEEE conference on Supercomputing (SC06)

[25] Shaw DE, Maragakis P, Lindorff-Larsen K, Piana S, Dror RO, et al (2010) Atomic-level characterization of the structural dynamics of proteins. Science 330: 341–6. 20947758 10.1126/science.1187409

[26] Zheng X, Diraviyam K (2007) Nucleotide effects on the structure and dynamics of actin. Biophysical Journal 93: 1277–1283. 17526584 10.1529/biophysj.107.109215 PMC1929039

[27] Pfaendtner J, Branduardi D, Parrinello M, Pollard TD, Voth GA (2009) Nucleotide-dependent conformational states of actin. Proceedings of the National Academy of Sciences of the United States of America 106: 12723–12728. 19620726 10.1073/pnas.0902092106 PMC2722336

[28] Tozzini V (2005) Coarse-grained models for proteins. Current Opinion in Structural Biology 15: 144–50. 15837171 10.1016/j.sbi.2005.02.005

[29] Go N (1983) Theoretical studies of protein folding. Annual Review of Biophysics and Bioengineering 12: 183–210. 10.1146/annurev.bb.12.060183.001151 6347038

[30] Baker D (2000) A surprising simplicity to protein folding. Nature 405: 39–42. 10811210 10.1038/35011000

[31] Koga N, Takada S (2001) Roles of native topology and chain-length scaling in protein folding: a simulation study with a Go-like model. Journal of Molecular Biology 313: 171–80. 11601854 10.1006/jmbi.2001.5037

[32] Takada S (1999) Go-ing for the prediction of protein folding mechanisms. Proceedings of the National Academy of Sciences of the United States of America 96: 11698–11700. 10518512 10.1073/pnas.96.21.11698 PMC33792

[33] Tirion M (1996) Large Amplitude Elastic Motions in Proteins from a Single-Parameter, Atomic Analysis. Physical Review Letters 77: 1905–1908. 10063201 10.1103/PhysRevLett.77.1905

[34] Hinsen K (1998) Analysis of domain motions by approximate normal mode calculations. Proteins 33: 417–429. 9829700 10.1002/(sici)1097-0134(19981115)33:3<417::aid-prot10>3.0.co;2-8

[35] Zheng W, Thirumalai D (2009) Coupling between normal modes drives protein conformational dynamics: illustrations using allosteric transitions in myosin II. Biophysical Journal 96: 2128–2137. 19289039 10.1016/j.bpj.2008.12.3897 PMC2717279

[36] Maragakis P, Karplus M (2005) Large amplitude conformational change in proteins explored with a plastic network model: adenylate kinase. Journal of Molecular Biology 352: 807–822. 16139299 10.1016/j.jmb.2005.07.031

[37] Miyashita O, Onuchic JN, Wolynes PG (2003) Nonlinear elasticity, proteinquakes, and the energy landscapes of functional transitions in proteins. Proceedings of the National Academy of Sciences of the United States of America 100: 12570–12575. 14566052 10.1073/pnas.2135471100 PMC240658

[38] Zheng W, Brooks BR (2005) Probing the local dynamics of nucleotide-binding pocket coupled to the global dynamics: myosin versus kinesin. Biophysical Journal 89: 167–178. 15879477 10.1529/biophysj.105.063305 PMC1366515

[39] Cui Q, Bahar I, editors. Normal Mode Analysis: Theory and Applications to Biological and Chemical Systems. Chapman & Hall/CRC

[40] Bahar I, Atilgan AR, Erman B (1997) Direct evaluation of thermal uctuations in proteins using a single-parameter harmonic potential. Folding & Design 2: 173–81. 9218955 10.1016/S1359-0278(97)00024-2

[41] Haliloglu T, Bahar I, Erman B (1997) Gaussian Dynamics of Folded Proteins. Physical Review Letters 79: 3090–3093.

[42] Doruker P, Atilgan AR, Bahar I (2000) Dynamics of proteins predicted by molecular dynamics simulations and analytical approaches: application to alpha-amylase inhibitor. Proteins 40: 512–24. 10861943

[43] Atilgan AR, Durell SR, Jernigan RL, Demirel MC, Keskin O, et al (2001) Anisotropy of uctuation dynamics of proteins with an elastic network model. Biophysical Journal 80: 505–15. 11159421 10.1016/S0006-3495(01)76033-X PMC1301252

[44] Tama F, Sanejouand YH (2001) Conformational change of proteins arising from normal mode calculations. Protein Engineering 14: 1–6. 11287673 10.1093/protein/14.1.1

[45] Liao JL, Beratan DN (2004) How does protein architecture facilitate the transduction of ATP chemical-bond energy into mechanical work? The cases of nitrogenase and ATP binding-cassette proteins. Biophysical Journal 87: 1369–1377. 15298939 10.1529/biophysj.103.038653 PMC1304475

[46] Chennubhotla C, Rader AJ, Yang LW, Bahar I (2005) Elastic network models for understanding biomolecular machinery: from enzymes to supramolecular assemblies. Physical Biology 2: S173–S180. 16280623 10.1088/1478-3975/2/4/S12

[47] Yang L, Song G, Jernigan RL (2007) How Well Can We Understand Large-Scale Protein Motions Using Normal Modes of Elastic Network Models? Biophysical Journal 93: 920–929. 17483178 10.1529/biophysj.106.095927 PMC1913142

[48] Piazza F, De Los Rios P, Sanejouand YH (2005) Slow Energy Relaxation of Macromolecules and Nanoclusters in Solution. Physical Review Letters 94: 145502. 15904073 10.1103/PhysRevLett.94.145502

[49] Togashi Y, Mikhailov AS (2007) Nonlinear Relaxation Dynamics in Elastic Networks and Design Principles of Molecular Machines. Proceedings of the National Academy of Sciences of the United States of America 104: 8697–8702. 17517661 10.1073/pnas.0702950104 PMC1868896

[50] Hayashi K, Takano M (2007) Violation of the uctuation-dissipation theorem in a protein system. Biophysical Journal 93: 895–901. 17496039 10.1529/biophysj.106.100487 PMC1913139

[51] Togashi Y, Yanagida T, Mikhailov AS (2010) Nonlinearity of Mechanochemical Motions in Motor Proteins. PLoS Computational Biology 6: e1000814. 20585540 10.1371/journal.pcbi.1000814 PMC2887453

[52] Flechsig H, Mikhailov AS (2010) Tracing entire operation cycles of molecular motor hepatitis C virus helicase in structurally resolved dynamical simulations. Proceedings of the National Academy of Sciences of the United States of America 107: 20875–20880. 21081697 10.1073/pnas.1014631107 PMC3000295

[53] Flechsig H, Popp D, Mikhailov AS (2011) In Silico Investigation of Conformational Motions in Superfamily 2 Helicase Proteins. PLoS ONE 6: e21809. 21829442 10.1371/journal.pone.0021809 PMC3139591

[54] Higo J, Umeyama H (1997) Protein dynamics determined by backbone conformation and atom packing. Protein Engineering 10: 373–380. 9194161 10.1093/protein/10.4.373

[55] Kim MK, Chirikjian GS, Jernigan RL (2002) Elastic models of conformational transitions in macromolecules. Journal of Molecular Graphics Modelling 21: 151–160. 12398345 10.1016/s1093-3263(02)00143-2

[56] Echeverria C, Togashi Y, Mikhailov AS, Kapral R (2011) A mesoscopic model for protein enzymatic dynamics in solution. Physical Chemistry Chemical Physics 13: 10527–10537. 21442113 10.1039/c1cp00003a

[57] Cressman A, Togashi Y, Mikhailov AS, Kapral R (2008) Mesoscale modeling of molecular machines: cyclic dynamics and hydrodynamical uctuations. Physical Review E 77: 050901. 10.1103/PhysRevE.77.050901 18643015

[58] Tirion MM, Ben-Avraham D (1993) Normal mode analysis of G-actin. Journal of Molecular Biology 230: 186–195. 8450535 10.1006/jmbi.1993.1135

[59] Chu JW, Voth GA (2007) Coarse-Grained Free Energy Functions for Studying Protein Conformational Changes: A Double-Well Network Model. Biophysical Journal 93: 3860–3871. 17704151 10.1529/biophysj.107.112060 PMC2084241

[60] Kabsch W, Mannherz HG, Suck D, Pai EF, Holmes KC (1990) Atomic structure of the actin: DNase I complex. Nature 347: 37–44. 2395459 10.1038/347037a0

[61] Chu JW, Voth GA (2006) Coarse-grained modeling of the actin filament derived from atomisticscale simulations. Biophysical Journal 90: 1572–82. 16361345 10.1529/biophysj.105.073924 PMC1367308

[62] Oguchi Y, Mikhailenko SV, Ohki T, Olivares AO, De La Cruz EM, et al (2008) Load-dependent ADP binding to myosins V and VI: implications for subunit coordination and function. Proceedings of the National Academy of Sciences of the United States of America 105: 7714–7719. 18509050 10.1073/pnas.0800564105 PMC2409399

[63] Yamaoka H, Matsushita S, Shimada Y, Adachi T (2011) Multiscale modeling and mechanics of filamentous actin cytoskeleton. Biomechanics and Modeling in Mechanobiology 11: 291–302. 21614531 10.1007/s10237-011-0317-z

[64] Janmey PA, Hvidt S, Oster GF, Lamb J, Stossel TP, et al (1990) Effect of ATP on actin filament stiffness. Nature 347: 95–99. 2168523 10.1038/347095a0

[65] Pollard TD, Borisy GG (2003) Cellular motility driven by assembly and disassembly of actin filaments. Cell 112: 453–465. 12600310 10.1016/s0092-8674(03)00120-x

[66] Atilgan C, Gerek ZN, Ozkan SB, Atilgan AR (2010) Manipulation of Conformational Change in Proteins by Single-Residue Perturbations. Biophysical Journal 99: 933–943. 20682272 10.1016/j.bpj.2010.05.020 PMC2913187

[67] Kitao A, Hirata F, N Go (1991) The effects of solvent on the conformation and the collective motions of protein: Normal mode analysis and molecular dynamics simulations of melittin in water and in vacuum. Chemical Physics 158: 447–472.

[68] Humphrey W, Dalke A, Schulten K (1996) VMD: visual molecular dynamics. Journal of Molecular Graphics 14: 33–38. 8744570 10.1016/0263-7855(96)00018-5

