# Supplementary figures and images for: Complex Intramolecular Mechanics of G-actin — An Elastic Network Study
Source: PLoS One. 2012 Oct 15;7(10):e45859. doi: 10.1371/journal.pone.0045859 (PMC3471905; doi:10.1371/journal.pone.0045859)

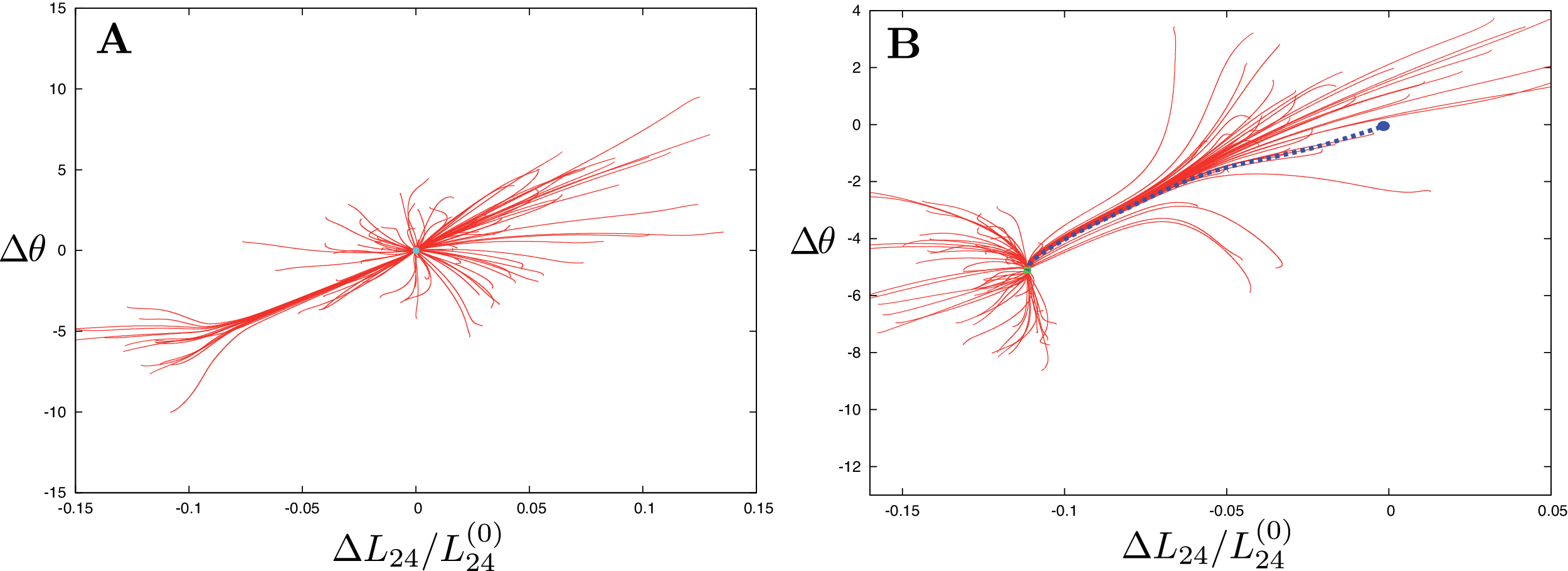

Supplement: Figure S1 — Patterns of relaxation trajectories for the elastic network of G-actin with the soft breakable links of strength Å2 in absence (A) and in the presence (B) of the ATP ligand. 100 relaxation trajectories starting from the initial conditions obtained by application of random static external forces to three sensitive residues in the NBP region are shown. The orientations of forces are random and their amplitudes are drawn at random from the interval between and Å. The blue curve shows the relaxation trajectory starting from the open equilibrium conformation of G-actin. As observed in panel A, the metastable state is absent when soft breakabale links are chosen. Nonetheless, the respective open protein conformations are easily visited as a result of perturbations. According to panel B, ligand binding leads to the appearance of a stable closed conformational state, whereas the open state of the protein becomes unstable. (TIFF) [file pone.0045859.s001.tif]
